# Microcystic macular edema in eyes with idiopathic epiretinal gliosis: A retrospective analysis before and after vitreomacular surgery: Short title: microcystic macular edema in idiopathic epiretinal gliosis

**DOI:** 10.1007/s00417-025-07006-1

**Published:** 2025-11-05

**Authors:** Denise Vogt, Julia Zimmermann, Adnan Kilani, Melih Parlak, Ricarda G. Schumann, Armin Wolf, Efstathios Vounotrypidis

**Affiliations:** 1 Department of Ophthalmology, University Hospital of Ulm, Ulm, Germany; 2 Munich Eye & Vascular Medicine Center, Munich, Germany

**Keywords:** Cystoid macular edema, Idiopathic epiretinal membrane, Microcystic macular edema membrane peeling, Optical coherence tomography, Vitrectomy, Vitreomacular interface vitreomaculopathy

## Abstract

**Purpose:**

To investigate incidence and clinical course of microcystic macular edema (MME) in eyes with idiopathic epiretinal membranes (iERM) before and after surgery.

**Methods:**

Of 1283 eyes that underwent vitrectomy with membrane peeling, 170 eyes were retrospectively analysed. All eyes had iERM and at least one postoperative control with retinal imaging. MME and cystoid macular edema (CME) were assessed by optical coherence tomography (OCT), along with demographic and clinical data. Correlations with functional outcomes were statistically significant at *p* < 0.05.

**Results:**

At baseline, 42 eyes (25%) had MME, 18 eyes (11%) had combined MME + CME and 110 eyes (64%) had no edema. The mean postoperative follow-up was 7.1 ± 5.9 months. All three groups showed significant improvement in visual acuity (VA), greatest in the no-edema group (*p* < 0.001). Incidence of MME increased with ERM severity (*p* = 0.033). At three months postoperatively, MME persisted in 32/159 eyes, resolved in 25, newly developed in 20, and was absent in 82/159 eyes, with a significant difference in VA between groups (*p* < 0.001). After 12 months, persistent MME reduced to 4/26 eyes, resolution of MME was seen in 6, new MME in 5 and 11/26 eyes still had no edema, with no significant VA between the groups (*p* = 0.206).

**Conclusion:**

Our study demonstrates that visual recovery occurred irrespective of MME status. In advanced iERM stages, MME was more frequently observed and associated with delayed visual improvement, particularly when it developed or persisted after surgery. Despite its early effects, MME does not seem to significantly affect long-term visual outcomes.

**Supplementary Information:**

The online version contains supplementary material available at 10.1007/s00417-025-07006-1.

## Key messages

*****What is known***:**

- Recent research has shown microcystic macular edema (MME) to occur more frequently in eyes with idiopathic epiretinal membrane (iERM).

*****What is new***:**

- Our study demonstrated that the incidence and short-term postoperative course of MME in iERM patients exhibit considerable clinical variability. The often unpredictable resolution may reflect underlying heterogeneity in disease mechanisms.
- The presence of MME was more frequent in advanced ERM stages and may be linked to delayed visual recovery, particularly when it develops or persists after surgery.
- MME appears to have limited impact on long-term visual function.

**Supplementary Information:**

The online version contains supplementary material available at 10.1007/s00417-025-07006-1.

## Introduction

Microcystic macular edema (MME) has recently been described to occur frequently in eyes with idiopathic epiretinal membrane (iERM) [1–6]. Identified by numerous small cystoid spaces within the inner nuclear layer (INL), MME typically does not affect the central foveal region [7, 8]. MME tends to appear in more advanced stages of iERM, and its features can resemble those of cystoid macular edema (CME), making it difficult to clearly distinguish between the two conditions [1]. Originally described as a neurodegenerative condition, ERM-related MME is now thought to be more closely linked to Müller cell dysfunction, which may explain its morphological and clinical features [1, 2, 4, 6].

Previous studies have reported that the presence of MME, whether preoperative or postoperative, is associated with poorer baseline visual acuity and worse functional outcomes [2–4, 9, 10]. MME has been identified as a poor prognostic factor for epiretinal membrane surgery, indicating chronicity of the ERM and damage to Müller cells [4]. It was demonstrated as the most common intraretinal cystoid space in iERMs and was significantly associated with preoperative visual impairment [3]. Additionally, microvascular leakage, detected via fluorescein angiography, has been associated with inner retinal deformity and poor visual recovery [9]. MME has been linked to delayed visual improvement and reduced effectiveness in controlling postoperative inflammation [2]. In contrast, it was recently published that the presence of MME was not strongly associated with visual acuity loss [1]. It was hypothesized that the impact of MME on visual acuity might appear marginal, potentially due to its predominantly paracentral location, which tends to spare the central foveal region [1]. Given its morphology and clinical characteristics, ERM-related MME has been proposed to be attributed to Müller cell disruption rather than a variant of a retrograde maculopathy [1, 11].

Since the impact of microcystic changes in iERM remains unclear, and conflicting reports exist regarding their effects on vision and surgical outcomes, the aim of this study was to investigate the incidence and clinical course of MME in eyes with iERM before and after macular surgery using retinal imaging with OCT.

## Materials and methods

From a cohort of 1283 eyes of 1140 patients that underwent pars plana vitrectomy (PPV) with peeling of the epiretinal membrane (ERM) and the inner limiting membrane (ILM) between 2021 and 2024, at the Department of Ophthalmology, University Hospital of Ulm, Ulm, Germany, this retrospective study included 170 eyes of 161 patients that were diagnosed with iERM and had a follow-up examination with retinal imaging at least three months after the operation.

Patients were excluded if they had history of concomitant ocular diseases e.g. other vitreomacular interface abnormalities, vitreoretinal diseases including full-thickness macular hole, glaucoma, diabetic retinopathy, high myopia, trauma, inflammatory or vascular diseases, if they presented other optic neuropathies affecting the eyes or MME associated with other retinal diseases and if they had undergone any prior intraocular surgery, except for uncomplicated phacoemulsification.

Medical records were analysed for age and sex at the time of surgery. Clinical data including best corrected visual acuity (BCVA) testing, lens status and intraocular pressure (IOP) was obtained before surgery and at 3 months, 6 months and ≥ 12 months after PPV, if available. For each case, the last documented follow-up (FUP) was also taken for analysis. Due to the retrospective nature of the study, a 6-week period was used for the baseline assessment, and a range of ± 6 weeks was permitted for each postoperative time point. BCVA was measured using objective refraction, followed by subjective refinement to determine the final correction, and was then converted to logarithm of the minimum angle of resolution (logMAR) scale for statistical analysis. The corresponding retinal imaging data was analysed.

After surgery, all patients received a combination of antibiotics (ofloxacin 3 mg/ml eye drops, three times daily over one week) and steroids (prednisolone 10 mg/ml eye drops, every hour daily over three days, then tapered every week). Standard escalating treatment of macular edema included additional topical non-steroidal anti-inflammatory NSAID eye drops (nepafenac 1 mg/ml three times daily), periocular steroid injections (40 mg triamcinolone) and the longer-lasting single sustained-release dexamethasone intravitreal implant (Ozurdex^®^, AbbVie).

The Institutional Review Board and the Ethics Committee of the University of Ulm, Ulm, Germany approved the study protocol (No 41/24). The study was conducted according to the tenets of the Declaration of Helsinki.

### Retinal imaging analysis

The retinal optical coherence tomography (OCT) images were obtained using either the SD-OCT (Spectralis, Heidelberg Engineering, Germany) or the Cirrus 6000 (Carl Zeiss Meditec Inc., Germany). The macular volume scans included in this study were routinely performed as part of standard clinical practice. Retinal layers’ segmentation was inspected by two retinal specialists (J.Z. and D.V.).

Idiopathic ERM was detected on cross-sectional OCT as a distinct, irregular, hyperreflective layer above the inner retinal surface and categorized based on a recognized four-stage OCT classification system, which was recently published by Govetto et al. [5]. According to this classification, stage 1 represents a mild iERM with a preserved foveal depression and well-defined retinal layers; stage 2 is an iERM with loss of foveal depression and well-defined retinal layers; stage 3 is an advanced iERM with loss of foveal depression and the presence of ectopic inner foveal layers (EIFL) over the central fovea, with well-defined retinal layers; stage 4 represents an advanced ERM with EIFL and disruption of all retinal layers [5].

The presence of MME and CME was carefully assessed in all available OCT scans preoperatively and postoperatively using established OCT criteria validated in previous studies [1, 2, 6]. MME was diagnosed as small and regular hyporeflective cystoid lesion within the inner nuclear layer only at the perifoveolar rim. CME appears as a grouping of cyst-like spaces of varying sizes and shapes, found in both the inner nuclear layer and the outer nuclear layer, including the central fovea. These cystoid spaces can fuse together, forming larger cavities, often accompanied by a localized detachment of the fovea [1]. To assess the course of MME, eyes without baseline MME were also evaluated postoperatively at each available visit for the presence of new MME, as indicated by the development of new small hyporeflective cystoid lesions within the inner nuclear layer, or eyes with baseline MME were evaluated for persistent or absent MME.

Additionally, the central macular thickness (CMT) was manually measured with caliper tool (in µm) using the built-in caliper function of the OCT device’s manufacturer-provided software at baseline and at each postoperative visit. Due to known systematic differences in thickness measurements between devices, data were analyzed separately by platform. Pre- and postoperative comparisons were conducted within each group, and outcomes were analysed stratified by OCT system. However, while both devices showed consistent trends and no significant differences were found between the measurements obtained from the two OCT devices (Spectralis, Heidelberg Engineering and Cirrus 6000, Zeiss), the data were pooled to report the results.

The presence of EIFL (present vs. absent), the presence of subfoveal cone bouquet abnormalities (CBA) (present vs. absent) and the state of the ellipsoid zone (EZ) (intact vs. disrupted) were also documented. EIFL refers to the abnormal persistence of inner retinal layers (such as the inner nuclear layer and inner plexiform layer) across the fovea, where they are normally absent [5]. CBA is a quantitative OCT biomarker measuring the angle formed at the foveal pit or retinal contour and reflects the severity of retinal distortion [5]. EZ integrity refers to the continuity of the ellipsoid zone (formerly called the IS/OS line) on OCT, which represents the photoreceptor inner segment/outer segment junction.

### Surgical procedure

All patients underwent a standard small gauge (23–25 g) pars plana vitrectomy with peeling of ILM and ERM using an end-gripping forceps. For intraoperative visualisation of the ILM a vital dye of 0.25 mg/ml solution of Brilliant Blue (Brilliant Peel; Fluoron GmbH, Neu-Ulm, Germany) or MEMBRANEBLUE-DUAL^®^ (D.O.R.C., Zuidland, The Netherlands) was used. At the end of surgery, the vitreous cavity was either filled with an endotamponade consisting of balanced salt solution (BSS, Bausch and Lomb, Germany) or purified air. In case of peripheral retinal breaks laser coagulation was applied accordingly.

### Statistical analysis

The data analysis was carried out using SPSS Statistics version 29.0.1.0 (IBM, Armonk, NY, USA). Summary statistics for continuous variables are presented as mean with standard deviation (SD), while frequency and percentage were calculated for categorical variables. Depending on the data type and distribution, appropriate tests were used. For paired comparisons of non-normally distributed continuous variables the Wilcoxon signed-rank test was applied. Independent group comparisons of non-parametric continuous data were conducted using the Mann–Whitney U test for two groups, and the Kruskal–Wallis test for comparisons involving more than two groups. Associations between categorical variables were evaluated with the Chi-square test, while trends across ordered categories were assessed using the linear-by-linear association test. For paired categorical data, the McNemar test and McNemar-Bowker test were utilized to determine differences between related proportions. MANOVA was used to test whether one or more categorical independent variables had a statistically significant effect on multiple, related continuous dependent variables simultaneously. A GEE model was used to analyze repeated categorical measurements over time while properly accounting for the within-patient correlations. The Cohen kappa coefficient was used to measure interobserver agreement for categorical variables. Differences were reported with 95% confidence intervals. A p-value below 0.05 was considered to indicate statistical significance. As this was a retrospective study a formal power calculation was not feasible.

At baseline and during FUP, the presence of MME and/or CME was categorized into 4 groups: (1) Preoperatively MME, (2) preoperatively CME, and (3) preoperatively combination of MME + CME, and (4) preoperatively no edema.

Additionally, we defined the following categorical subgroups to analyze MME patterns over longitudinal FUP: (1) Persistent MME: baseline MME + postoperative MME (2) New onset MME: baseline no MME + postoperative MME, (3) Resolution of MME: baseline MME + postoperative no MME, (4) No MME: Absence of baseline or postoperative MME.

Patients who were lost to follow-up were excluded from the respective analyses at each timepoint. No imputation methods (e.g., last observation carried forward or multiple imputation) were applied. All longitudinal analyses were conducted using available-case data. As such, the number of eyes included in each analysis may vary due to incomplete follow-up data. The potential for selection bias arising from differential loss to follow-up is acknowledged and discussed.

## Results

This study included a total of 170 eyes of 161 patients, 81 females (50%) and 80 male (50%), corresponding to 85 right (50%) and 85 left eyes (50%). The patients’ mean age at time of surgery was 71.4 ± 7.4 years (range, 43–86 years). The mean axial length of the eyes was 23.79 ± 1.25 mm (range, 19.06–25.85 mm). Of the 104 phakic eyes (61%), combined vitrectomy with ERM and ILM peeling and cataract surgery was performed in 96 eyes (92%). During FUP, one eye had additional cataract surgery. At last FUP, seven eyes (4%) were phakic and 163 eyes (96%) pseudophakic.

Two masked graders independently evaluated all the OCT scans of the included ERMs to obtain the retinal imaging data. The interobserver agreement was 88–93%, with a Cohen kappa coefficient of 0.81–0.89 indicating almost perfect agreement.

### Subgroup differences in eyes with MME and/or CME categorized at baseline

The detailed parameters are provided in Table 1. At baseline 42 of 170 eyes (25%) presented with MME, 18 of 170 eyes (11%) showed combination of MME + CME and 110 of 170 eyes (64%) had no edema. No eye presented with CME alone before macular surgery. The FUP was comparable between the three groups: MME group: 7.1 ± 5.7 months (range, 2–23 months), MME + CME group: 8.9 ± 6.7 months (range, 2–27 months), group without edema: 6.8 ± 5.0 months (range, 2–27 months).

**Table 1:** Clinical and retinal imaging data at baseline and last documented follow-up (FUP) were analyzed across three subgroups defined by baseline edema status: microcystic macular edema (MME), MME with cystoid macular edema (CME), and no edema

	MME 42 eyes				MME + CME 18 eyes				No edema 110 eyes			
Analysis of demographical and clinical data												
Age [years]	68 ± 8 SD				71 ± 7 SD				71 ± 7 SD			
Sex [m/f] (%)	18/24 (42%/57%)				10/8 (56%/44%)				57/53 (52%/48%)			
Eye [right/left] (%)	22/20 (52%/48%)				10/8 (56%/44%)				53/57 (48%/52%)			
Axial length [mm]	23.70 ± 1.19 SD				23.80 ± 1.24 SD				23.82 ± 1.28			
Intraocular pressure [mmHg] Preoperatively Last FUP	16.0 ± 2.6 SD 15.2 ± 3.0 SD				15.0 ± 3.0 SD 15.8 ± 4.0 SD				14.5 ± 3.0 SD 15.1 ± 3.1 SD			
Lens status [phakic/pseudophakic] (%) Preoperatively Last FUP	24/18 (57%/43%) 5/37 (12%/88%)				10/8 (56%/44%) 0/18 (0%/100%)				70/40 (63%/36%) 2/108 (2%/98%)			
BCVA [logMAR] Preoperatively Phakic Pseudophakic Last FUP Phakic Pseudophakic	0.47 ± 0.30 logMAR^1^ 0.52 ± 0.34 logMAR^2^ 0.41 ± 0.21 logMAR^3^ 0.26 ± 0.26 logMAR^1^ 0.29 ± 0.31 logMAR^2^ 0.21 ± 0.18 logMAR^3^			^1^*p*<0.001^a^* ^2^*p*<0.001^a^* ^3^*p*=0.002^a^*	0.46 ± 0.18 logMAR^1^ 0.48 ± 0.16 logMAR^2^ 0.43 ± 0.21 logMAR^3^ 0.21 ± 0.18 logMAR^1^ 0.22 ± 0.19 logMAR^2^ 0.20 ± 0.17 logMAR^3^	^1^*p*=0.002^a^* ^2^*p*<0.002^a^* ^3^*p*=0.017^a^*			0.39 ± 0.18 logMAR^1^ 0.38 ± 0.18 logMAR^2^ 0.39 ± 0.18 logMAR^3^ 0.21 ± 0.15 logMAR^1^ 0.18 ± 0.16 logMAR^2^ 0.24 ± 0.14 logMAR^3^		^1^*p*<0.001^a^* ^2^*p*<0.001^a^* ^3^*p*<0.001^a^*	
Follow-up (FUP) [months]	7.1 ± 5.7 SD				8.9 ± 6.7 SD				6.8 ± 5.0 SD			
OCT analysis												
ERM classification Stage 1, (%) Stage 2, (%) Stage 3, (%) Stage 4, (%)	*p* = 0.033^b*^											
	0 (0%) 24 (57%) 14 (33%) 4 (10%)				0 (0%) 11 (61%) 6 (33%) 1 (6%)					0 (0%) 82 (75%) 26 (23%) 2 (2%)		
CMT [in µm] Preoperatively Last FUP	476 ± 97 SD 411 ± 49 SD	*p <* 0.001^a^*			440 ± 68 SD 371 ± 47 SD			*p =* 0.002^a^*	453 ± 78 SD 399 ± 48 SD		*p <* 0.001^a^*	
CBA [yes/no] (%) Preoperatively Last FUP	15/27 (36%/64%) 9/33 (21%/78%)	*p =* 0.018^c^*			12/6 (67%/33%) 4/14 (22%/78%)		*p =* 0.021^c^*		58/52 (53%/47%) 32/78 (29%/71%)		*p <* 0.001^c^*	
EIFL [yes/no] (%) Preoperatively Last FUP	15/27 (36%/64) 9/33 (21%/79%)		*p =* 0.039^c^*		7/11 (39%/61%) 0/18 (0%/100%)		*p =* 0.016^c^*		34/76 (31%/69%) 12/98 (11%/89%)		*p <* 0.001^c^*	
EZ defects [yes/no] (%) Preoperatively Last FUP	15/27 (36%/64%) 12/30 (29%/71%)		*P* = 0.196^c^		9/9 (50%/50%) 4/14 (22%/78%)		*p =* 0.031^c^*		39/71 (36%64%) 36/74 (33%/67%)			*p* = 0.44^c^

*FUP* follow-up, *BCVA* best-corrected visual acuity, *OCT* optical coherence tomography, *ERM* epiretinal membrane, *CMT* central macular thickness, *CBA* cone bouquet abnormalities, *EIFL* ectopic inner foveal layers, EZ defects, ellipsoid zone defects

^a^Wilcoxon test, ^b^Chi-square test, ^c^Mc Nemar test

*statistically significant

The McNemar–Bowker test revealed a significant asymmetry in the distribution of macular edema types between baseline and last FUP (*p* = 0.045). Descriptive analysis showed that a number of patients improved from combined macular edema (CME + MME) at baseline to no edema or isolated MME at last follow-up, while no patients worsened from no edema to combined CME + MME. This suggests a general trend toward improvement in edema status post-treatment.

Each group demonstrated a statistically significant improvement in BCVA following surgery (MME group: *p* < 0.001, MME + CME group: *p* = 0.002, group without edema: *p* < 0.001; Wilcoxon test). When comparing the degree of visual improvement across the groups, no significant differences were detected (*p* = 0.249; Kruskal-Wallis test). Table 1 provides the BCVA values, categorised according to lens status. Of note, lens status was similar between the groups at both baseline and last FUP (*p* = 0.668 and *p* = 0.919; Chi-square test). There was no significant difference in visual acuity change (ΔVA from baseline) between the three MME subgroups in relation to lens status grouping at baseline (MME group: *p* = 0.857; MME + CME group: *p* = 0.497; no edema group: *p* = 0.117, Mann-Whitney U test). In addition, a factorial MANOVA was conducted to examine the effects of the type of macular edema and the type of surgery on the change in BCVA from baseline to the last follow-up. The analysis revealed that neither the preoperative type of macular edema (Hotelling’s trace, *p* = 0.264) nor the type of surgery (Hotelling’s trace, *p* = 0.473) had a statistically significant effect on the change in BCVA.”

Across all three groups, OCT imaging revealed stage 2 ERM as the most frequently observed stage, followed in descending order by stages 3 and 4. A significant association was observed between ERM stage and the presence of MME (*p* = 0.033; Chi-square test). The Linear-by-Linear Association was also significant (*p* = 0.011), suggesting that the likelihood of MME increases with ERM severity. Surgical treatment led to a statistically significant reduction of CMT in each group (MME group: *p* < 0.001; MME + CME group: *p* = 0.002; group without edema: *p* < 0.001; Wilcoxon test). There was no statistically significant difference in the change of CMT among the different groups (*p* = 0.517, Kruskal-Wallis test).

The presence of CBA, EIFL, and EZ defects did not differ significantly across the groups at baseline and last FUP (each *p* > 0.05; Chi-square test). This is illustrated by the graphs in the supplementary data. Similarly, no statistically significant association was found between ERM stage and the presence of CBA within any of the groups (each *p* > 0.05; Fisher’s exact test). A significant difference was observed in both the change in CBA status and the EIFL status from baseline to the last FUP in all three groups (MME group: CBA *p* = 0.180, EIFL *p* = 0.039; MME + CME group: CBA *p* = 0.021, EIFL *p* = 0.016; group without edema: both *p* < 0.001; McNemar test). The change in EZ defect status from baseline to the last FUP was not statistically different in all three groups (MME group: *p* > 0.05; MME + CME group: *p* = 0.031; group without edema: *p* > 0.05; McNemar test).

### Clinical outcomes at baseline and postoperative time points (Follow-up comparison)

Table 3. presents a summary of the OCT parameters and BCVA assessed at baseline and at each FUP. The detailed analysis of postoperative treatment for different macular edema states is given in Table 2 for the various time points.

**Table 2:** Analysis of postoperative treatment for different macular edema States

	Macular edema status	Number of eyes (%)	Therapy				
			Therapy (yes/no)	NSAID eye drops (yes/no)	Steroids eye drops (yes/no)	Steroids parabulbar (yes/no)	Dexamethasone implant (yes/no)
**3 Months** **FUP** *N* = 159 eyes	MME only	48 (28%)	23/25	20/28	9/39	7/41	1/47
	CME only	3 (2%)	2/1	0/3	1/2	0/3	1/2
	both MME and CME	5 (3%)	5/0	3/2	1/4	3/2	0/5
	No edema	103 (61%)	30/73	14/89	25/78	0/103	0/103
			*p* = 0.002^a^*	*p* < 0.001^a^*	*p* = 0.853^a^	*p* < 0.001^a^*	*p* < 0.001^a^*
**6 Months** **FUP** *N* = 71 eyes	MME only	14 (19%)	10/4	8/6	4/10	2/12	2/12
	CME only	0 (0%)	--	--	--	--	--
	both MME and CME	3 (4%)	2/1	1/2	0/3	0/3	1/2
	No edema	54 (76%)	4/50	3/51	1/53	0/54	0/54
			*p* < 0.001^a^*	*p* < 0.001^a^*	*p* = 0.002^a^*	*p* = 0.014^a^*	*p* = 0.002^a^*
**12 months** **FUP** *N* = 55 eyes	MME only	18 (33%)	8/10	8/10	3/15	1/17	1/17
	CME only	0 (0%)	--	--	--	--	--
	both MME and CME	1 (2%)	0/1	0/1	0/1	0/1	0/1
	No edema	36 (65%)	0/36	0/36	0/36	0/36	0/36
			*p* < 0.001^a^*	*p* < 0.001^a^*	*p* = 0.173^a^	*p* = 0.351^a^	*p* = 0.351^a^

*FUP* follow-up, *MME* microcystic macular edema, *CME* cystoid macular edema, *NSAID* Non-steroidal anti-inflammatory drugs

^a^Chi-square test/Fisher’s exact test,

*, statistically significant

**Table 3.** Analysis of postoperative treatment for different macular edema states

		OCT data					Functional data
		Macular edema	CMT [µm]	CBA [yes/no] (%)	EIFL [yes/no] (%)	EZ defects [yes/no] (%)	BCVA [logMAR]
Baseline *N* = 170 eyes	Total MME only (%) CME only (%) both MME and CME (%) no edema	170 (100%) 42 (25%) 0 (0%) 18 (11%) 110 (64%)	457 ± 82 SD 475 ± 97 SD -- 440 ± 68 SD 453 ± 78 SD	85/85 (50%/50%) 15/27 -- 12/6 58/52	56/114(33%/67%) 15/27 -- 7/11 34/76	63/107 (63%/37%) 15/27 -- 9/9 39/71	0.41 ± 0.22 SD 0.47 ± 0.30 SD -- 0.46 ± 0.18 SD 0.39 ± 0.18 SD
			*p* = 0.517^a^	*p* = 0.056^c^	*p* = 0.726^c^	*p* = 0.485^c^	*p* = 0.249^a^
FUP 3 months *N* = 159 eyes	Total MME only (%) CME only (%) both MME and CME (%) no edema	159 (100%) 48 (28%) 3 (2%) 5 (3%) 103 (61%)	446 ± 81 SD 467 ± 83 SD^+^ 417 ± 127 SD 457 ± 76 SD 455 ± 82 SD+	59/100 (37%/63%) 17/31 1/2 5/0 36/67	31/128(19%/81%) 13/35 2/1 2/3 14/89	67/92 (58%/42%) 20/28 1/2 3/2 43/60	0.25 ± 0.21 SD 0.32 ± 0.24 SD^+^ 0.43 ± 0.49 SD 0.54 ± 0.26 SD 0.20 ± 0.17 SD^+^
			*p* < 0.001^a^* *p* < 0.001^b^*	*p* = 0.033^c^	*p* = 0.022^c^	*p* = 0.859^c^	*p* < 0.001^a^* *p* < 0.001^b^*
FUP 6 months *N* = 71 eyes	Total MME only (%) CME only (%) both MME and CME (%) no edema	71 (100%) 14 (19%) 0 (0%) 3 (4%) 54 (76%)	396 ± 50 SD 442 ± 39 SD^+^ -- 395 ± 67 SD 384 ± 46 SD^+^	24/47 (34%/66%) 7/7 -- 2/1 15/39	6/65 (8%/92%) 4/10 -- 0/3 2/52	19/52 (27%/73%) 4/10 -- 2/1 13/41	0.24 ± 0.20 SD 0.31 ± 0.32 SD^+^ -- 0.33 ± 0.06 SD 0.21 ± 0.16 SD^+^
			*p* < 0.001^a^* *p* < 0.001^b^*	*p* = 0.138^c^	*p* = 0.010^c^*	*p* = 0.264^c^	*p* = 0.156^a^ *p* < 0.001^b^*
FUP ≥ 12 months *N* = 55 eyes	Total MME only (%) CME only (%) both MME and CME (%) no edema	55 (100%) 18 (33%) 0 (0%) 1 (2%) 36 (65%)	391 ± 47 SD 417 ± 48 SD ^a^ -- 451 377 ± 41 SD ^a^	10/45 (18%/82%) 2/16 -- 1/0 7/29	5/50(9%/91%) 4/14 -- 1/0 5/50	7/48 (13%/87%) 5/13 -- 0/1 2/34	0.23 ± 0.21 SD 0.27 ± 0.23 SD -- 0.40 0.20 ± 0.20 SD
			*p* = 0.003^b^*	*p* = 0.076^c^	*p* = 0.061^c^	*p* = 0.064^c^	*p* = 0.333^b^

*FUP* follow-up, *OCT* optical coherence tomography, *MME* microcystic macular edema, *CME* cystoid macular edema, *CMT* central macular thickness, *CBA* cone bouquet abnormalities, *EIFL* ectopic inner foveal layers, *EZ* defects, ellipsoid zone defects, *BCVA* best-corrected visual acuity

^a^Kruskal Wallis test, ^b^Mann-Whitney U test, ^c^Chi-square test/Fisher’s exact test,

*, statistically significant

At 3-months FUP, eyes with MME had significantly worse BCVA and greater CMT compared to eyes without edema at this timepoint (*p* < 0.001 and *p* = 0.001; Mann-Whitney U test). There was also statistically significant difference in BCVA and CMT, when performing an intergroup comparison between all groups at this time point (each *p* < 0.001; Kruskal-Wallis test). The BCVA improvement from baseline to 3-months FUP was statistically significant in eyes with MME and eyes without edema (MME group: *p* = 0.013; MME + CME group: *p* = 0.285; CME group: *p* = 0.785; group without edema: *p* < 0.001; Wilcoxon test). CMT significantly decreased only in eyes with MME (*p* = 0.003) and in eyes without edema (*p* < 0.001), but not in the MME + CME (*p* = 0.225) or CME group (*p* = 1.000) (Wilcoxon test). The presence of CBA and EIFL differed significantly between the groups (CBA *p* = 0.033 and EIFL *p* = 0.022; Fisher’s exact test). Additionally, a significant relationship was found between the presence of MME and the use of local NSAID eye drops (*p* = 0.005) and topical steroids (*p* < 0.001) (Chi-square test). There was no significant asymmetry in changes of MME/CME status between baseline and 3-months-FUP (McNemar-Bowker test, *p* = 0.188).

At 6-months FUP, there was no statistically significant difference in BCVA between the groups, but eyes without edema showed a significantly greater reduction in CMT compared to eyes with MME (MME vs. no edema: BCVA *p* = 0.244 and CMT *p* < 0.001; Mann-Whitney U test; all eyes: BCVA *p* = 0.156 and CMT *p* < 0.001; Kruskal-Wallis test.). BCVA and CMT changed significantly during FUP in eyes with MME (*p* < 0.001 and *p* = 0.031) and in eyes without edema (*p* < 0.001 and *p* < 0.001), while the difference was not significant in the MME + CME group (each *p* > 0.05) (Wilcoxon Test). The presence of EIFL differed significantly between the groups (*p* = 0.010; Fisher’s exact test). The use of local NSAID eye drops (*p* < 0.001), topical steroids (*p* = 0.002), parabulbar steroids (*p* = 0.014) and dexamethasone implant (*p* = 0.012) was found significantly more frequent in eyes with MME and/or CME (Chi-square test). There was a significant association between baseline and 6-months FUP MME/CME status (Chi-square test, *p* = 0.007), with most eyes showing stability or improvement, although the McNemar–Bowker test indicated only borderline evidence for asymmetry in changes (*p* = 0.056).

After 12 months of FUP, there was no statistically significant difference in BCVA, but CMT differed significantly between the MME and no edema groups (BCVA *p* = 0.213 and CMT *p* = 0.003; Mann Whitney U test). BCVA improvement and CMT reduction was significantly from baseline in both groups (MME group: BCVA *p* < 0.001 and CMT *p* = 0.004; group without edema: BCVA *p* < 0.001 and CMT *p* < 0.001; Wilcoxon test). There were no significant differences between the groups in the presence of CBA, EIFL or EZ defects (each *p* > 0.05; Chi-square test:). Presence of MME correlated significantly with use of NSAID eye drops (*p* < 0.001; Chi-Square-Test). The McNemar–Bowker test revealed a significant asymmetry in changes of MME/CME status between baseline and follow-up (*p* = 0.045).

Of note, the 3 eyes (2%) that exhibited CME alone at the 3-month follow-up after macular surgery were each examined once more and showed no signs of edema at their final follow-up visit. Two out of the three eyes were treated, with one eye receiving a dexamethasone implant and the other just steroid eye drops (Table 2).

### Subgroup analysis of change in MME during Follow-Up (Longitudinal Comparison)

Follow-up data were available for 159 eyes at 3 months. Of these, 65 eyes were additionally followed at 6 months, and 26 eyes had complete FUP at 3, 6, and a minimum of 12 months. Given the limited number of cases, all eyes exhibiting MME were categorized as ‘MME’ in the analysis, irrespective of whether concurrent CME was present or not. The complete data set is presented in Table 4..

**Table 4.** Subgroup analysis of change in microcystic macular edema (MME) during follow-up (FUP)

		Code	Total number (%)	Presence of edema[at baseline]			BCVA[logMAR]	P-value	CMT[µm]	P-value
				MME	MME + CME	No edema				
**FUP 3 months** *N* = 159 eyes	Persistent MME (since baseline)	1–1	32 (20%)^1^	26 (16%)	6 (3%)	0	0.37 ± 0.29 SD	*p* = 0.001^a^*	426 ± 49 SD	*p* = 0.007^a^*
	Resolution of baseline edema after surgery	1 − 0	25 (16%)^2^	16 (10%)	9 (6%)	0	0.21 ± 0.22 SD		393 ± 46 SD	
	Development of postoperative MME	0–1	20 (13%)	0	0	20 (13%)	0.30 ± 0.14 SD		434 ± 51 SD	
	No edema (since baseline)	0–0	82 (51%)	0	0	82 (52%)	0.20 ± 0.14 SD		408 ± 48 SD	
**FUP 6 months** *N* = 65 eyes	Persistent MME (since baseline)	1-1-1	10 (15%)	8 (12%)	2 (3%)	0	0.31 ± 0.37 SD	*p* = 0.537^a^	428 ± 36 SD*	*p* = 0.001^a^*
	Resolution of MME Early Resolution of baseline MME Late Resolution of baseline MME Resolution of new postOP MME	1-0-0 1-1-0 0–1-0	17(26%) 9 (14%) 6 (9%)^3^ 2 (3%)	5 (8%) 5 (8%) 0	4 (6%) 1(2%) 0	0 0 2 (3%)	0.22 ± 0.18 SD		377 ± 50 SD	
	Development of MME Persistent new post OP MME (since FUP 3 mo) New MME at FUP 6 mo Recurrent MME at FUP 6 mo	0–1-1 0-0-1 1-0-1	7 (11%) 6 (9%) 0 (0%) 1 (2%)^4^	0 -- 0	0 -- 1 (2%)	6 (9%) -- 0	0.30 ± 0.16 SD		451 ± 43 SD	
	No edema (since baseline)	0-0-0	31 (48%)	0	0	31 (47%)	0.22 ± 0.16 SD		386 ± 43 SD*	
**FUP ≥ 12 months** *N* = 26 eyes	Persistent MME (since baseline)	1-1-1-1	4 (15%)	3 (12%)	1 (4%)	0	0.40 ± 0.40 SD	*p* = 0.206^a^	426 ± 24 SD	*p* = 0.011^a^*
	Resolution of MME Resolution of baseline MME Resolution of post OP MME Resolution of baseline + post OP MME	1-0-0-0 0–1-0-0/0–1-1-0/0-0-1-0 1-1-0-0/1-0-1-0	2 (8%) -- 4 (15%)	1 (4%) -- 3 (11%)	1 (4%) -- 1 (4%)	0 -- 0	−0.50 ± 0.07 SD 0.28 ± 0.17 SD		337 ± 39 SD 334 ± 29 SD	
	Development of MME Persistent new post OP MME (since FUP 3 mo) New MME at FUP 6 mo New MME at FUP 12 mo	0–1-1-1 0-0-1-1 0-0-0–1	5 (19%) -- --	0 -- --	0 -- --	5 (19%) -- --	0.16 ± 0.11 SD		428 ± 52 SD	
	No edema (since baseline)	0-0-0-0	11 (42%)	0	0	11 (42%)	0.22 ± 0.18 SD		395 ± 45 SD	

*FUP* follow-up, *MME* microcystic macular edema, *CME* cystoid macular edema, *BCVA* best-corrected visual acuity, *CMT* central macular thickness

^a^Kruskal-Wallis test,

*. statistically significant

^1^Among them, 2 eyes with additional CME; ^2^Among them, 2 eyes with additional CME; ^3^Among them,1 eye with additional CME;^4^This eye had additional CME

At 3-months FUP, 32 eyes (20%) had persistent MME. In 25 eyes (16%) with MME at baseline, the MME resolved postoperatively. In contrast, 20 eyes (13%) without MME at baseline had new MME documented postoperatively. No MME was seen in 82 eyes (51%). There was a significant difference in follow-up BCVA and CMT across MME outcome groups (*p* = 0.001 and *p* = 0.007; Kruskal–Wallis test). Eyes with persistent or newly developed MME showed significantly worse BCVA and greater CMT compared to those with resolved or no MME.

At the 6-month FUP, 10 eyes (15%) exhibited persistent MME. Resolution of MME was observed in 17 eyes (26%). Among these, the majority—9 eyes (14%)—showed resolution at the first postoperative visit, while 6 eyes (9%) demonstrated late resolution by the 6-month FUP. Additionally, resolution of newly developed postoperative MME was noted in 2 eyes (3%). The occurrence of MME was noted in 7 eyes (11%). Of these, 6 eyes (9%) exhibited a newly developed, persistent MME since the 3-months FUP. One eye that had MME at baseline experienced a recurrence at 6 months, after the edema had initially resolved following surgery. Although CMT was statistically different between the MME outcome groups (*p* = 0.001; Kruskal–Wallis test), no significant differences in six months FUP BCVA were observed among the different MME outcome groups (*p* = 0.537; Kruskal–Wallis test).

At 12-months FUP, in 4 of the 26 eyes (15%) MME persisted throughout the follow-up. Resolution of MME was noted in 6 of 26 eyes (23%). Among them, in 2 eyes (8%) with baseline MME complete resolution of the edema was observed after surgery. Resolution of MME at baseline and after surgery was documented in 4 of 26 eyes (15%). In 5 eyes (19%) without edema first showed MME postoperatively, which was present in all 5 eyes 3 months after surgery. A total of 11 eyes (42%) remained free of edema at any time during the entire follow-up. There was no significant difference in BCVA between the groups (*p* < 0.206), but CMT was significant lower in eyes without MME compared to eyes with MME (*p* = 0.011, Kruskal-Wallis test).

The GEE revealed a statistically significant change in the distribution of macular edema types across the four time points (*p* = 0.012). Descriptive analysis showed that at 3 months a few more eyes had any type of macular edema compared with baseline. This indicates a worsening of edema status in the early follow-up period. However, the distribution of edema types at the 6-month and 12-month follow-up was not significantly different from the baseline, suggesting a general trend of improvement in the edema status back to baseline levels after the 3-month mark.

## Discussion

Our study shows that visual recovery occurred regardless of type of edema. All groups demonstrated significant postoperative improvements in BCVA and reductions in CMT, with no overall differences in visual gains between groups. Preoperative MME occurrence increased with advancing ERM stage. At 3 months, eyes with MME showed worse BCVA despite general improvements, and no significant CMT reduction was seen in the MME + CME group. Longitudinal analysis confirmed that persistent or newly developed MME was associated with worse visual and anatomical outcomes compared to eyes with resolved or no MME. However, by 12 months, BCVA differences between groups were no longer significant, although CMT remained higher in the MME group. Longitudinal data highlighted that the persistence of MME was associated with greater preoperative CMT, while resolution—especially of both baseline and postoperative MME—was linked to more favorable anatomical outcomes. Structural markers (CBA, EIFL, EZ defects) showed no significant intergroup differences at twelve months FUP.

Several studies have identified MME as a negative prognostic factor in ERM surgery, linking it to poorer pre- and postoperative visual outcomes, reduced visual recovery, and less frequent resolution after surgery [2–4, 9, 10, 12]. However, recent data suggest that MME has only marginal impact on both preoperative and postoperative visual acuity with no strong association with visual acuity loss [1]. Govetto and colleagues reported a heterogeneous but generally improving postoperative course in iERM eyes with MME [1]. Similarly, Dysli et al. found no significant difference in visual acuity between eyes with and without postoperative MME [11]. Our study supports these findings, as eyes in all groups improved significantly after surgery, regardless of the presence of MME. Of note, MME was linked to worse short-term postoperative outcomes, but its impact on visual acuity appears to lessen over time, with recovery comparable to non-MME eyes by 12 months.

There is no data to show that the use of NSAIDs or steroid therapy leads to a significant improvement in visual acuity in eyes with MME. Nevertheless, the presence of MME appears to influence ophthalmologists to extend the duration of NSAID eye drop treatment in our study. Despite treatment, MME resolved in some cases but persisted in others, reflecting individual differences in healing responses. Variability in the extent of retinal damage, inflammation control, and intrinsic recovery capacity may explain the diverse visual outcomes and heterogeneity seen across studies. Reduced effectiveness in controlling inflammation after surgery has been reported in previous studies [2, 4]. This vulnerability may be further exacerbated in advanced stages of ERM, where the membrane tends to be thicker and exerts greater tractional force on the retinal tissue. Surgical factors such as differences in surgical technique, completeness of membrane peeling and intraoperative trauma may also influence postoperative recovery and MME behavior. However, in the present study, all cases underwent both membrane and internal limiting membrane (ILM) peeling. Similarly, the study by Dysli et al. found no significant differences in outcomes related to the use of additional ILM peeling [11]. Güler et al. also reported the same incidence of MME in patients undergoing ERM alone versus ERM and ILM peeling [13]. The frequent appearance of MME in ERMs, contrasted with its rarity in macular holes, suggests that factors beyond ILM peeling contribute to its pathogenesis [1].

The pathogenesis of MME is not fully understood, but it is believed to involve several interrelated mechanisms affecting the inner retinal layers, particularly the Müller cells. The increasing presence of MME with higher ERM stages in our study supports its role as a marker of disease severity and mechanical retinal traction. Recently, Govetto et al. concluded, based on a combination of clinical data and histopathological evidence, that MME does not represent a form of retrograde maculopathy [1]. MME may reflect Müller cell disruption or tractional damage to inner retinal layers [14]. Under normal conditions, fluid moves from the superficial to deep capillary plexus and is absorbed by Müller cells via AQP-4 channels [15]. Since both the deep capillary network and Müller cell bodies lie in the inner nuclear layer, vitreomacular traction can impair fluid regulation, leading to Müller cell degeneration and retinal fluid accumulation [1, 5, 14]. In advanced ERMs (stages 3 and 4), stronger and longer lasting tractional forces may more severely disrupt Müller cell function, potentially leading to irreversible chronic changes and greater susceptibility to mechanical stress, thus possibly explaining the higher incidence of MME in these cases [1, 2]. The development of new MME following membrane peeling may be attributed to ganglion cell injury, which subsequently impairs Müller cell function, particularly their role in fluid regulation and homeostasis [11, 13]. Against this theory, MME was originally described in the context of neurodegenerative diseases, and microcysts were thought to develop as a result of retrograde trans-synaptic degeneration, where the loss of ganglion cells triggers the degeneration of bipolar cells in the inner nuclear layer, resulting in the formation of fluid-filled spaces or cavities within the retina [7, 16].

This study has several limitations, primarily related to its retrospective design. The use of pre-existing clinical data may have introduced selection bias and include high variability, as only patients who sought care and had at least one follow-up visit with OCT imaging were included. In Germany, due to the well-established structure of outpatient ophthalmological care—particularly in rural areas—patients are often managed locally for follow-up. It is therefore plausible that patients with poorer macular morphology or suboptimal surgical outcomes are more likely to return for hospital-based follow-up. Conversely, patients with unremarkable postoperative findings may prefer to continue care closer to home. As a result, a substantial number of patients may not be referred back to the hospital for further evaluation. Consequently, a significant proportion of patients who had not been referred back to hospital for further evaluation were excluded from this study. Furthermore, missing or inconsistently documented variables could have affected the accuracy and completeness of the analysis. For instance, information on underlying systemic comorbidities (e.g. diabetes without diabetic retinopathy, hypertension and neurodegeneration) was not included. In addition, eyes imaged with different devices (Heidelberg Engineering and ZEISS) were included, potentially introducing variability. Certain imaging techniques, such as en face OCT, were not routinely performed in the pre- or postoperative period, and therefore could not be assessed in this analysis. Furthermore, the presence of macular edema was evaluated qualitatively rather than through standardized quantitative measurements over time. Finally, the lack of adjustment for lens status and the potential influence of concurrent cataract surgery on visual acuity outcomes, as well as the absence of subjective functional parameters such as metamorphopsia, represent limitations of the study. Some of these aspects—particularly subjective measures—may not have been assessable due to the retrospective study design. Additionally, non-significant results may reflect limited statistical power rather than a true absence of effect. We also emphasize that the use of a conventional p-value threshold (*p* < 0.05) should not be interpreted in isolation, and we underscore the need for adequately powered prospective studies to validate these findings.

In conclusion, our results show that the incidence and postoperative course of MME in iERM show a diverse and heterogeneous clinical pattern during short-term follow-up, with resolution often unpredictable, possibly indicating heterogeneous sign of condition or diseases. The presence of MME was more frequent in advanced ERM stages and may be linked to delayed visual recovery, particularly when it develops or persists after surgery. Nonetheless, MME appears to have limited impact on long-term visual function. These findings might suggest that most of the MME does not represent chronic and refractory changes. Further studies are needed to understand the pathogenesis of MME and to investigate therapeutic options.

## Supplementary Information

Below is the link to the electronic supplementary material.

Supplementary Material 1 (JPG 1.12 MB)
