# Supplementary figures and images for: Microcystic macular edema in eyes with idiopathic epiretinal gliosis: A retrospective analysis before and after vitreomacular surgery: Short title: microcystic macular edema in idiopathic epiretinal gliosis
Source: Graefes Arch Clin Exp Ophthalmol. 2025 Nov 5;264(2):393–405. doi: 10.1007/s00417-025-07006-1 (PMC12923396; doi:10.1007/s00417-025-07006-1)

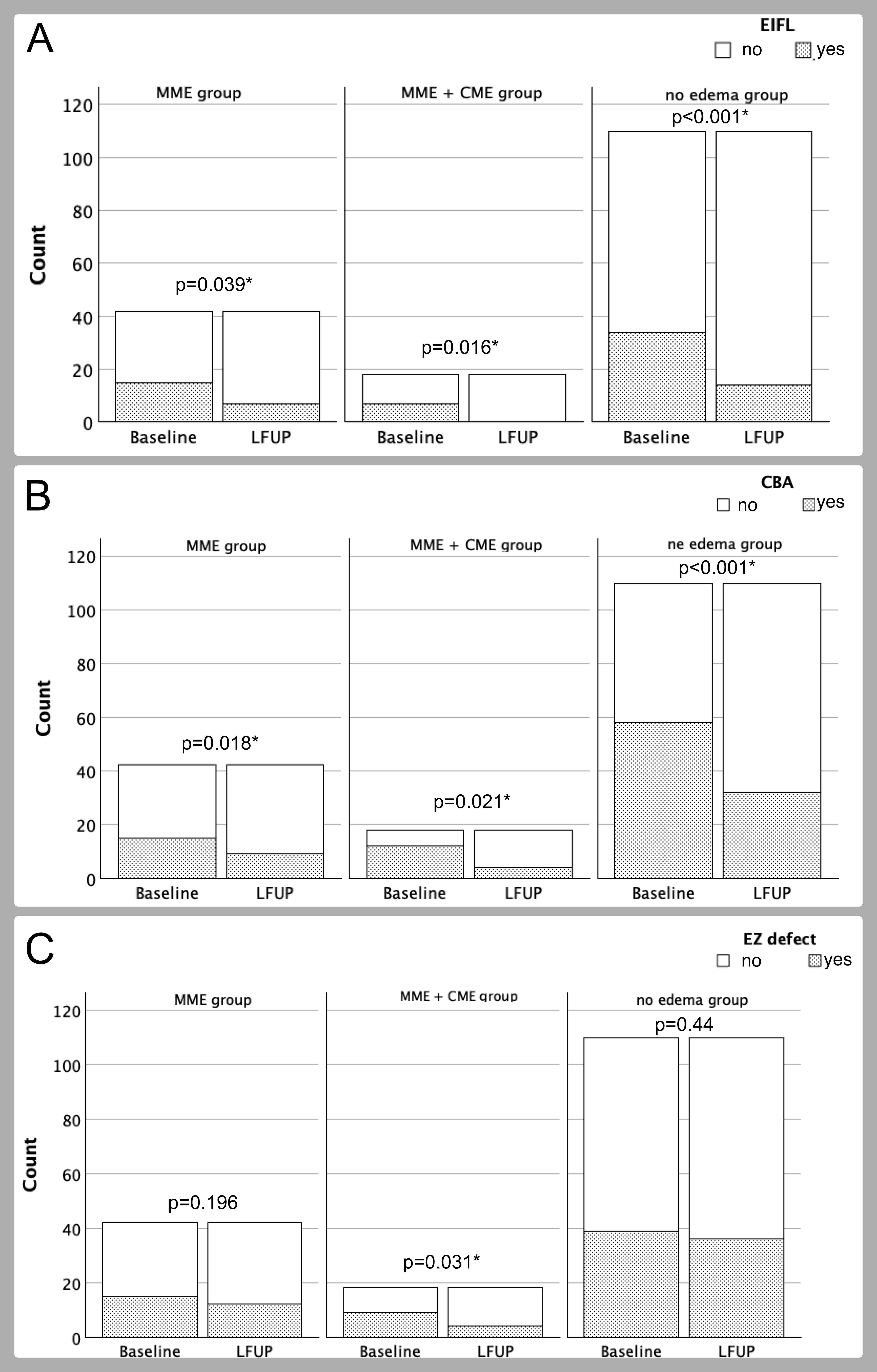

Supplement: Supplementary file 1 — Supplementary Material 1 (JPG 1.12 MB) [file 417_2025_7006_MOESM1_ESM.jpg]
